# A randomized placebo-controlled trial of convalescent plasma for adults hospitalized with COVID-19 pneumonia

**DOI:** 10.1038/s41598-022-19629-z

**Published:** 2022-09-30

**Authors:** Louise Thorlacius-Ussing, Patrick Terrence Brooks, Henrik Nielsen, Bitten Aagaard Jensen, Lothar Wiese, Susanne Gjørup Sækmose, Stine Johnsen, Mikkel Gybel-Brask, Isik S. Johansen, Mie Topholm Bruun, Nina Breinholdt Stærke, Lars Østergaard, Christian Erikstrup, Sisse Rye Ostrowski, Keld Mikkelsen Homburg, Jørgen Georgsen, Susan Mikkelsen, Håkon Sandholdt, Cæcilie Leding, Nichlas Hovmand, Clara Lundetoft Clausen, Michaela Tinggaard, Karen Brorup Heje Pedersen, Katrine Kjær Iversen, Sandra Tingsgård, Simone Bastrup Israelsen, Thomas Benfield

**Affiliations:** 1 Center of Research and Disruption of Infectious Diseases, Department of Infectious Diseases, Copenhagen University Hospital –Amager and Hvidovre, Hvidovre, Denmark; 2 Department of Clinical Immunology, Copenhagen University Hospital –Rigshospitalet, Copenhagen, Denmark; 3 Department of Infectious Diseases, Aalborg University Hospital, Aalborg, Denmark; 4 Department of Clinical Medicine, Aalborg University, Aalborg, Denmark; 5 Department of Clinical Immunology, Aalborg University Hospital, Aalborg, Denmark; 6 Department of Infectious Diseases, Zealand University Hospital, Roskilde, Denmark; 7 Department of Clinical Immunology, Zealand University Hospital, Køge, Denmark; 8 Department of Respiratory Medicine, Copenhagen University Hospital –Bispebjerg and Frederiksberg, Copenhagen, Denmark; 9 Department of Infectious Diseases, Odense University Hospital, Odense, Denmark; 10 Department of Clinical Immunology, Odense University Hospital, Odense, Denmark; 11 Department of Infectious Diseases, Aarhus University Hospital, Aarhus, Denmark; 12 Department of Clinical Immunology, Aarhus University Hospital, Aarhus, Denmark; 13 Institute of Clinical Medicine, University of Copenhagen, Copenhagen, Denmark

**Keywords:** Medical research, Infectious diseases, Viral infection

## Abstract

Passive immunotherapy with convalescent plasma may be the only available agent during the early phases of a pandemic. Here, we report safety and efficacy of high-titer convalescent plasma for COVID-19 pneumonia. Double-blinded randomized multicenter placebo-controlled trial of adult patients hospitalized with COVID-19 pneumonia. The intervention was COVID-19 convalescent plasma and placebo was saline allocated 2:1. The primary outcome was clinical status 14 days after the intervention evaluated on a clinical ordinal scale. The trial was registered at ClinicalTrials.Gov, NCT04345289, 14/04/2020. The CCAP-2 trial was terminated prematurely due to futility. Of 147 patients randomized, we included 144 patients in the modified intention-to-treat population. The ordinal clinical status 14 days post-intervention was comparable between treatment groups (odds ratio (OR) 1.41, 95% confidence interval (CI) 0.72–2.09). Results were consistent when evaluating clinical progression on an individual level 14 days after intervention (OR 1.09; 95% CI 0.46–1.73). No significant differences in length of hospital stay, admission to ICU, frequency of severe adverse events or all-cause mortality during follow-up were found between the intervention and the placebo group. Infusion of convalescent plasma did not influence clinical progression, survival or length of hospitalization in patients with COVID-19 pneumonia.

## Introduction

Early in a pandemic, such as the ongoing outbreak of the coronavirus disease-19 (COVID-19) caused by severe acute respiratory syndrome coronavirus 2 (SARS-CoV-2), it is unlikely that specific therapeutic agents will be available. Plasma collected from a patient convalescent from SARS-CoV-2 infection contains neutralizing antibodies against SARS-CoV-2 with a possible therapeutic role for passive immunotherapy. Passive immunotherapy has been used in the past century for respiratory infections caused by SARS-CoV-1, middle eastern respiratory syndrome (MERS), and influenza virus with uncertain benefit^1–3^.

Several randomized controlled trials of COVID-19 convalescent plasma were initiated during the first year of the pandemic, including this trial^4^. The largest trial of convalescent plasma, the Randomized Evaluation of COVID-19 Therapy (RECOVERY) Trial, enrolled more than 11,000 participants. In RECOVERY, high-titer convalescent plasma did not improve survival or other clinical outcomes. These results were published as a pre-print on March 10, 2021^5^. Based on these results, our Data and Safety Monitoring Board (DSMB) recommended stopping enrolment to our trial, the CREDID COVID-19 Adaptive Platform (CCAP)-2 trial, on March 16, 2021 due to a high likelihood of futility.

The objective of our trial was to evaluate the efficacy and safety of convalescent plasma compared with placebo for hospitalized patients with COVID-19 pneumonia. Here we report the modified intention-to-treat (mITT) analysis of the primary and secondary outcomes of the individuals who received the allocated trial intervention in CCAP-2.

## Methods

### Study design

The CREDID COIVD-19 Adaptive Platform (CCAP)2 trial was a randomized double-blinded, placebo-controlled, multicenter trial conducted at six clinical sites in Denmark. Eligible participants were randomly assigned in a 2:1 ratio to receive convalescent plasma or placebo. The trial protocol was approved by the Ethical Committee of the Capital Region of Denmark (record no. H-20025317) and is available in the Appendix. Written informed consent was obtained from all participants, and the trial was conducted in accordance with the principles stated in the Declaration of Helsinki. The authors take full responsibility for the design and conduct of the trial and vouch for the accuracy and completeness of the data, the analysis of the data, and the adherence of the trial to the protocol. The trial was registered at ClinicalTrials.Gov, NCT04345289, 14/04/2020.

### Participants

At each participating site, hospitalized adults (≥ 18 years) were eligible for enrolment if they were positive for SARS-CoV-2 by a reverse-transcriptase–polymerase-chain-reaction (RT-PCR) of a respiratory tract sample, had evidence of pneumonia as determined by radiologically confirmed pulmonary infiltrate and/or peripheral oxygen saturation (SaO2) below 94% while breathing ambient air, and a negative pregnancy test for women of childbearing potential.

Exclusion criteria of the study were I) Pregnancy or breastfeeding, II) Participation in other drug clinical trials, III) Any serious medical condition or abnormality of clinical laboratory test, that in the investigator’s judgement precluded the patients safe participation or completion of the study, IV) Progression to death was imminent and inevitable within the next 24 h, irrespectively of the provision of the treatment.

### Randomization and masking

Eligible patients underwent treatment allocation and concealment through the randomization program Research Electronic Data Capture (REDCap) and were assigned in a 2:1 ratio to receive either COVID-19 convalescent plasma (600 mL) or placebo (600 mL of 0.9% saline solution) in addition to standard of care treatment^6,7^. Opaque covers were used to conceal the intravenous lines and infusion bags (Supplemental Fig. S1). The participants, the clinical team, the data collectors, and the outcome adjudicators were all blinded to the treatment assignments. Patients could receive remdesivir, glucocorticoids and anticoagulants according to the standard of care at the providing health care institution.

### Trial procedures

Plasma donors were males between 18 and 65 years of age with a previously confirmed SARS-CoV-2 infection (presence of SARS-CoV-2 nucleic acid by PCR or anti-SARS-CoV-2 immunoglobulin), who had been asymptomatic for at least 4 weeks at time of plasmapheresis, and met general criteria for plasma donor eligibility. Male donors were used in order to reduce the risk of Transfusion-related acute lung injury (TRALI) due to anti-human leukocyte antigen antibodies that are more frequent in female donors. Plasma donors were either registered blood donors or previous patients diagnosed with COVID-19. All plasma donors provided informed consent prior to plasmapheresis as per local blood donor protocol. Plasma was obtained by plasmapheresis at the blood establishment associated with the public hospital services in the five Danish Regions according to standard procedures and national guidelines^8^. At each apheresis, 600 mL of plasma was collected and split into two units of 300 mL. Convalescent plasma was required to have a minimum SARS-CoV-2 immunoglobulin (Ig) G antibody titer of > 3.0 (EUROIMMUN AG, Lübeck, Germany). Convalescent plasma for each patient was predominantly from a single donor. Both units of 300 mL were transfused within 24 h.

### Outcomes

The primary outcome of the trial was changed from a comparison of survival on day 28 to clinical status on day 14. The amendment was proposed on January 15, 2021, after discussions between the DSMB and sponsor due to low study accrual and a nationwide decline in new cases. The trial was paused until the amendment was approved on January 23, 2021. The original primary outcome became the key secondary end point. The revised primary outcome of clinical status 14 days after intervention was defined by one of seven mutually exclusive ordinal categories on an adapted version of the World Health Organization (WHO) clinical scale: (1) death; (2) hospitalized, in intensive care requiring Extracorporeal Membrane Oxygenation (ECMO) or mechanical ventilation; (3) hospitalized, on non-invasive ventilation or high-flow oxygen device; (4) hospitalized, requiring supplemental oxygen; (5) hospitalized, not requiring supplemental oxygen; (6) not hospitalized, limitations on activities and/or requiring home oxygen; and (7) not hospitalized, no limitations on activities^9^. On day 14, the primary outcome was determined as the worst of the seven categories which the participant fulfilled.

Secondary outcomes were: Frequency of infusion-related adverse events, frequency of severe-adverse-events, all-cause mortality or need of invasive mechanical ventilation up to day 28, ventilator-free days to day 28, organ failure-free days to day 28, duration of intensive care unit (ICU) stay, mortality at days 7, 14, 21, 28, and 90, length of hospital stay and duration of supplemental oxygen.

After the trial intervention, patients were followed in person during the hospital admission and by telephone after hospital discharge. Details regarding data collection, patient follow-up, randomization, the data blinding and masking process, and plasma donation, collection, processing, and storage are provided in the Supplementary.

### Statistical analysis

With the original primary outcome, the trial was designed to enroll 1,100 participants. The change in primary outcome permitted a more realistic sample size with use of a widely accepted clinical outcome used for COVID-19 trials. The amendment was proposed when 111 of 1100 participants according to the original sample size calculation had been enrolled. In the revised version, the trial was designed to enroll 530 patients (353 in the plasma group and 177 in the placebo group) assuming a specific severity distribution at day 14, including an expected mortality rate within 14 days of 6.5% in the intervention group and 10% in the placebo group (Supplemental Table S1). These assumptions were based on data from studies conducted in the early phase of the pandemic^10,11^. We calculated that this sample size would provide 80% power to detect a proportional odds ratio of 1.6 for plasma as compared with placebo on the clinical ordinal scale at the 0.05 (two-sided) level of significance. The analysis was performed using R version 3.6.0 (R Foundation for Statistical Computing, Vienna, Austria). Details regarding the overall statistical analysis, interim analysis, and unblinding criteria are provided in the Supplementary Material.

## Results

### Patients

**Figure 1:**
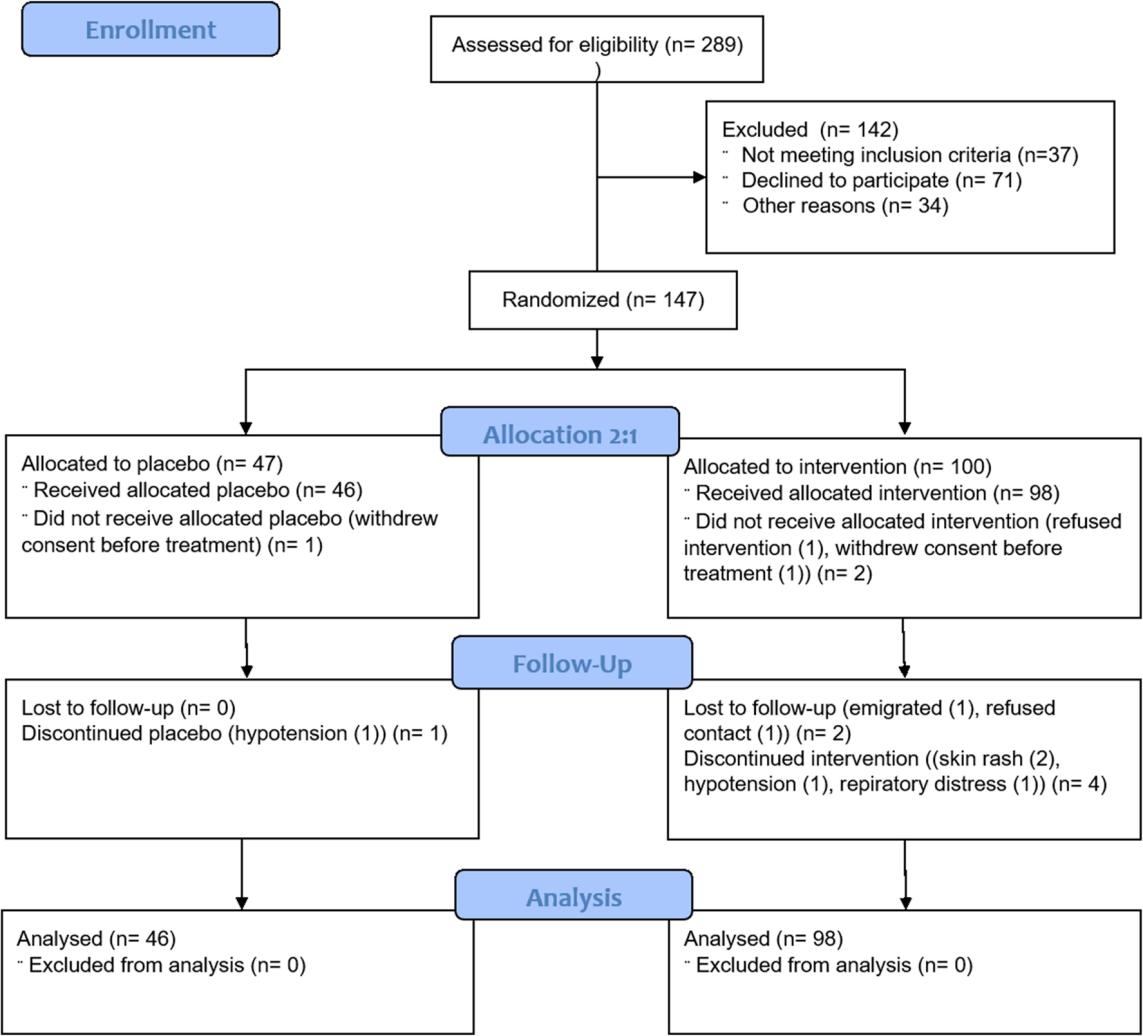
Enrollment, Randomization, and Analysis Populations.

Between June 13, 2020 and March 16, 2021, a total of 289 patients were assessed for eligibility, and 147 patients were enrolled. Three patients withdrew informed consent before receiving their treatment intervention. Consequently, 98 patients received convalescent plasma and 46 participants received placebo (Fig. 1) and were included in the mITT analysis. Two participants, who were discharged on day 2 and 3, respectively, refused contact during follow up but were alive on day 90. Their ordinal scale status was conservatively set to 6 on day 14.

The baseline characteristics of patients included in the mITT analysis are shown in Table 1. The median age of the patient population was 65 years (interquartile range [IQR] 55–75); 72% of the patients were men, and 77% had a coexisting comorbidity at trial entry. The median time from the onset of COVID-19 symptoms to enrollment was 11 days (IQR 8–13). At baseline, patients had a median respiratory rate of 20 per min (IQR 18–22) and a median oxygen saturation of 94% (IQR 92–96). Median values of lymphocytes, D-dimer and C-reactive protein were 0.8 × 10^9^ (IQR 0.7–1.2) cells/L, 0.8 (IQR 0.5–1.4) Units/L and 64.5 (IQR 36.2–115.5) mg/L, respectively. The majority (92%) of the patients received supplemental oxygen, of whom 5% required mechanical ventilation, 33% high-flow oxygen or non-invasive ventilation, and 54% low-flow oxygen. Most patients were treated with remdesivir (87%), dexamethasone (90%), and anticoagulation (55%) at the time of trial entry.

**Table 1:** Baseline characteristics of patients included in the modified intention to treat analysis.

	Convalescent plasma (n = 98)	Placebo (n = 46)
**Age, years, median (IQR)**	66 (53–75)	64 (57–73)
**Female sex, n (%)**	28 (29)	12(26)
**Race, n (%)**		
Caucasian	67 (68)	35 (78)
Middle Eastern	26 (27)	9 (20)
Other	5 (5)	1 (2)
Not reported	0 (0)	1 (2)
**Body mass index, kg/m**^**2**^**, median (IQR)**	29 (26–33)	28 (25–33)
Not reported	25	10
**Duration of symptoms before randomization, days, median (IQR)**	11 (8–13)	10 (8–13)
**Comorbidity, n (%)**	75 (77)	35 (76)
Arterial hypertension	27 (28)	14 (30)
Cardiovascular disease	38 (39)	18 (39)
Diabetes	25 (26)	7 (15)
Chronic pulmonary disease	11 (11)	5 (11)
Cancer	9 (9)	1 (2)
Cerebral disease	17 (17)	8 (17)
Other	9 (9)	4 (9)
**Vitals, median (IQR)**		
Temperature, ℃	36.5 (36.1–36.8)	36.6 (36.1–36.9)
Glasgow Coma Score	15 (15–15)	15 (15–15)
Respiratory rate per minute	20 (18–22)	19 (18–20)
Oxygen saturation, %	94 (92–96)	94 (92–96)
Heart rate per minute	76 (67–84)	69 (62–78)
Mean arterial pressure, mmHg	79 (75–91)	87 (84–99)
**Laboratory values, median (IQR)**		
B-Hemoglobin, mmol/L	8.3 (7.4–8.7)	8.4 (7.3–8.9)
B-Lymphocyte cell count, × 10^9^/L	0.9 (0.7–1.2)	0.8 (0.6–1.3)
B-Platelet count, × 10^9^/L	261 (180–328)	241 (183–305)
P-D-dimer, Units/L	0.8 (0.5–1.4)	0.6 (0.5–1.3)
Estimated Glomerular Filtration Rate, L/minute	90 (74–90)	90 (74–90)
P-alanineaminotransferase, Units/L	43 (27–63)	39 (31–56)
P-Lactate dehydrogenase, Units/L	1.1 (0.9–1.4)	1.2 (0.9–1.8)
P–C-reactive protein, mg/L	64 (37–120)	67 (33–108)
P-Procalcitonin, µg/L	0.2 (0.1–0.2)	0.1 (0.1–0.2)
**Treatment during trial, n (%)**		
Remdesivir	87 (89)	37 (84)
Corticosteroids	87 (89)	41 (93)
Anticoagulation	52 (53)	27 (61)
**Clinical progression scale, n (%)**		
Mechanical ventilation	4 (4)	3 (7)
Hospitalized; NIV or HF	41 (42)	9 (19)
Hospitalized; requiring supplemental oxygen	46 (47)	30 (65)
Hospitalized; not requiring supplemental oxygen	7 (7)	4 (9)

*IQR* interquartile range, *NIV* non-invasive ventilation, *HF* high-flow, *P* plasma, *B* blood.

**Figure 2:**
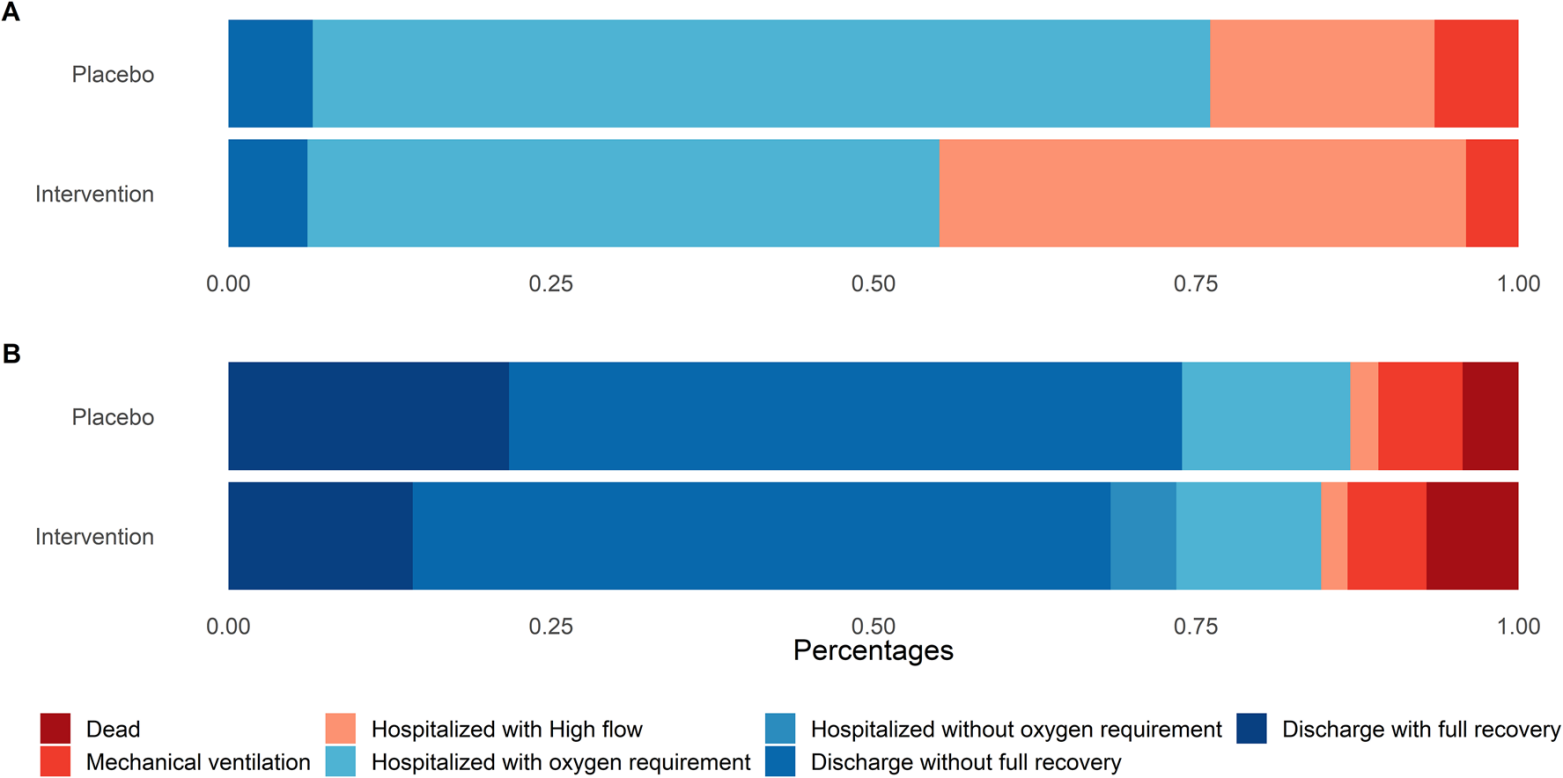
Clinical progression scale at baseline (**A**) and by 14 days (**B**).

At baseline, patients receiving convalescent plasma more often required high-flow oxygen or non-invasive ventilation than patients who received placebo (Table 1 and Fig. 2). Patients receiving convalescent plasma and placebo were comparable on all other baseline variables.

### Intervention and placebo administration

The median EUROIMMUN SARS-CoV-2 IgG titer of the infused convalescent plasma was 5.2 (IQR 3.6 to 6.6). All participants in the placebo group received the full saline infusion whereas 93 of 96 in the intervention group received the full amount of convalescent plasma; one discontinued due to hypotension and two because of a skin rash.

### Primary outcome

**Figure 3:**
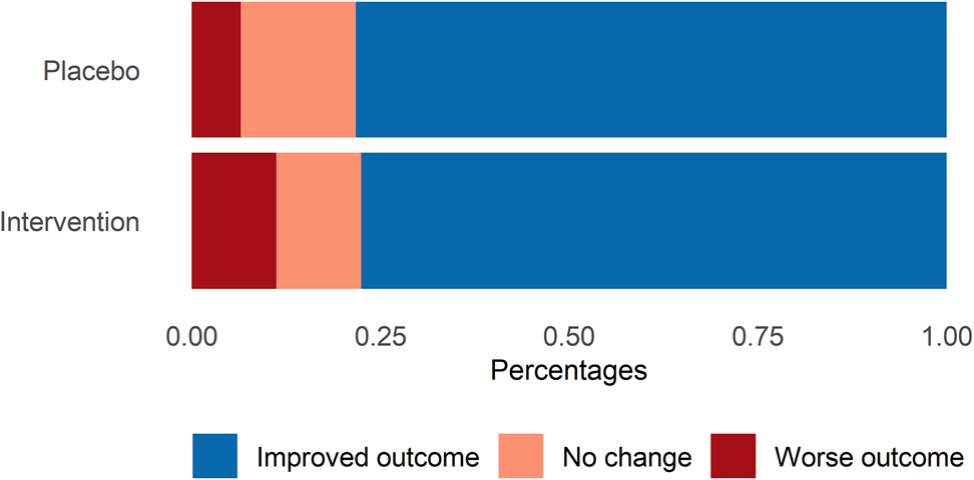
Clinical progression scale on individual level by 14 days.

The distribution of clinical outcomes on day 14 according to the clinical ordinal scale was comparable between the convalescent plasma group and the placebo group, odds ratio (OR) 1.41; 95% confidence interval [CI], 0.72 to 2.09; P = 0.321) for worse outcome (Table 2 and Fig. 2). In a post hoc analysis, individual clinical progression by day 14 did not differ according to treatment group (OR 1.09; 95% CI, 0.46–1.73) for convalescent plasma vs. placebo (Fig. 3).

**Table 2:** Worst scores for Covid-19 severity at 14 days according to a 7-category ordinal scale.

Ordinal scale, day 14	Convalescent plasma (n = 98)	Placebo (n = 46)
1. Death	7 (7%)	2 (4%)
2. Hospitalized, requiring extracorporeal membrane oxygenation or mechanical ventilation	6 (6%)	3 (7%)
3. Hospitalized, on non-invasive ventilation or high-flow oxygen device	2 (2%)	1 (2%)
4. Hospitalized, requiring supplemental oxygen	11 (11%)	6 (13%)
5. Hospitalized, not requiring supplemental oxygen	5 (5%)	0 (0%)
6. Not hospitalized with limitation in activity	53 (54%)	24 (52%)
7. Not hospitalized without limitation in activity	14 (15%)	10 (22%)

### Secondary outcomes

The 90-day mortality was 16% (15 of 96 patients) in the convalescent plasma group and 9% (4 of 46) in the placebo group (Table 3). No significant between-group differences in survival was seen at day 7, 14, 28, or 90. The median time from enrollment to death was 11 days^5–14^ in the convalescent plasma group and 9 days^8–12^ in the placebo group. Thirteen of 16 deaths in the convalescent plasma group and four out of four deaths in the placebo group occurred during hospitalization for COVID-19. ICU admission and mechanical ventilation was required by 14% and 7%, respectively, in the convalescent plasma group and 10% and 5%, respectively, in the placebo group. Duration of ICU admission was seven days (IQR 4–18) for patients in the convalescent plasma group compared to 14 days (IQR 8–20) for patients in the placebo group. No differences were noted for ventilator-free days, organ-failure-free days or oxygen supply days between groups. The median time from enrollment to hospital discharge was 6 days (IQR 3–13) in the convalescent plasma group and four days (IQR 2–8) in the placebo group.

**Table 3:** Secondary outcomes.

	Convalescent plasma (n = 98)	Placebo group (n = 46)	Odds ratio (95% CI) or median difference (95% CI)
Deaths through 7 days (%)	5/98 (5)	0/46 (0)	_
Deaths through 14 days (%)	7/98 (7)	2/46 (4)	1.69 (0.34–8.48)
Deaths through 21 days (%)	11/98 (11)	3/46 (7)	1.81 (0.48–6.84)
Deaths through 28 days (%)	12/98 (12)	3/46 (7)	2.00 (0.54–7.47)
Deaths through 90 days (%)	15/98 (15)	4/46 (9)	1.90 (0.59–6.08)
SAE inclusive deaths through 28 days (%)	25/98 (26)	8/46 (17)	1.66 (0.67–3.95)
Time to death, median (IQR)	11 (5–14)	9 (8–12)	2 (− 9 to 7)
Deaths during hospitalization for Covid-19 (%)	13/98 (14)	4/46 (9)	1.61 (0.49–5.23)
Mechanical ventilation through 28 days (%)*	6/94 (6)	2/43 (5)	1.40 (0.27–7.23)
Ventilator-free days through 28 days, median (IQR)	28 (28–28)	28 (28–28)	0 (0–0)
Organ failure through 28 days (%)	9/98 (9)	3/46 (7)	1.45 (0.37–5.63)
Organ failure free days through 28 day, median (IQR)	28 (28–28)	28 (28–28)	0 (0–0)
Admission to ICU through 28 days (%)**	12/89 (13)	4/39 (10)	1.36 (0.42–4.53)
Duration of ICU admission, days, median (IQR)	7 (4–18)	14 (8–20)	− 7 (− 17 to 7)
Duration of oxygen supply days, median (IQR)***	6 (3–15)	6 (3–14)	0 (− 3.0 to 4.0)
Length of hospital stay, days, median (IQR)	6 (3–13)	6 (3–8)	0 (− 2 to 3.5)

*SAE* Severe adverse events, *IQR* interquartile range, *ICU* Intensive care unit, *CI* confidence interval.

*Patients on mechanical ventilation at randomization not included.

**Patients admitted to the ICU at randomization not included.

***Patients not requiring oxygen supply not included.

### Safety results

Infusion-related adverse events (hypotension (n = 1), rash (n = 2) and respiratory distress (n = 1)) were more common in the convalescent plasma group (4.2%; 4 of 96 patients) than in the placebo group (hypotension (n = 1) (2.2%; 1 of 46 patients). Although statistically insignificant, a higher frequency of severe adverse events was reported for the convalescent plasma group compared to the placebo group (26% vs 17%). Reported adverse and severe adverse events during follow-up are provided in Supplemental Tables S2 and S3.

## Discussion

The CCAP-2 trial was stopped after a third of participants had been recruited based on a recommendation from the DSMB due to futility. In this prematurely terminated trial, we found that hospitalized patients with COVID-19 pneumonia who received convalescent plasma did not have better clinical outcomes at day 14 than those who received placebo. Similarly, the rates of death through 90 days as well as other secondary outcomes were comparable in both treatment arms. The safety profile was comparable with convalescent plasma and placebo.

Numerically, there was a higher proportion of individuals with more severe ordinal outcomes at day 14 and more severe adverse events and deaths at all time points in the convalescent plasma group in our study. The differences were not statistically significantly different at any point. By chance, participants in the convalescent plasma group had significantly more severe disease at inclusion by the ordinal scale. It is likely, that this explains the differences in outcomes between groups because an analysis of individual outcomes showed an OR of 1.05 (95% CI, 0.41–1.69) for clinical progression by day 14 (convalescent plasma versus placebo).

The uncertainty of the 28-day mortality estimate in this study encompasses the results reported by the RECOVERY trial and an accompanying metaanalysis of 11 trials including an additional 1,973 individuals to the 11,258 participants of RECOVERY, showing no beneficial effects of treatment with convalescent plasma in relation to mortality at day 28^5^. The RECOVERY trial did not report clinical progression by day 14.

Convalescent plasma is administered to increase levels of neutralizing antibodies in the recipient before the recipient has mounted a humoral response. Trial data suggests that administration of plasma within 72 h of symptom onset may prevent progression to severe respiratory disease for outpatients with mild disease, while administration to high-risk outpatients within 7 days of symptom onset in another trial did not prevent disease progression^12,13^. A randomized trial from Spain (n = 350) reported a benefit of convalescent plasma given within 7 days of symptom onset for a composite endpoint including respiratory status and death at day 28^14^. Early administration of high-titer plasma was associated with a lower risk of death in an observational trial^15^. In our trial, participants were enrolled into the trial within a median of 2 days (IQR 1.8–4.0) of admission to hospital and regardless of length of symptoms. The median duration of symptoms was 11 days (IQR 8–11) at randomization in our trial and, thus, comparable to most other trials that did not show a benefit of convalescent plasma^5,16,17^. Therefore, convalescent plasma may still play a role if administered early and before the development of native antibodies. To further investigate a strategy of early administration in a controlled trial, participants would need to be recruited prior to hospital admission and preferably also have their antibody levels determined at baseline.

Any effect of convalescent plasma is most likely correlated with the titers of neutralizing antibodies, and failure to demonstrate a beneficial effect of convalescent plasma in prior studies could potentially be a consequence of use of plasma with low titers of neutralizing antibodies. In this study, we used convalescent plasma with a EUROIMMUN IgG titer > 3.0, which correlated closely (R^2^ = 0.91) to an IgG titer of 15 measured by the Ortho Vitros assay^18^. Hence the convalescent plasma used in this study qualified as high-titer plasma in accordance with the US FDA Emergency Use Authorization for COVID-19 convalescent plasma of August, 2020, which required an anti-SARS-CoV-2 IgG titer of 12 measured by the Ortho Vitros assay. The median EUROIMMUN IgG titer was in fact > 5 in this trial and, as such, it is unlikely that the null result observed in this study was related to insufficient titers of neutralizing antibodies.

### Study amendments

The trial design was amended on January 15, 2020. The amendment included changing the primary outcome from a comparison of survival on day 28 to clinical status on day 14. This was done for several reasons. Accrual to the study had been low initially due to few hospitalizations between June and October of 2020, hospitals were overwhelmed in November and December of 2020 due to a steep increase in hospitalizations leading to transfer of study resources at several study sites (in fact, only three of six sites enrolled a significant number of participants), there was a temporary shortage of convalescent plasma in the Capital Region of Denmark (the region had enrolled two thirds of participants) in January 2021 due to limited resources, and a waning epidemic after January 2021with fewer hospitalizations for COVID-19. The advice from our Data Safety and Monitoring Board was that enrollment of 1000 participants wasn’t feasible. Additionally, an increasing number of COVID-19 trials applied the revised WHO clinical ordinal scale as the primary outcome to evaluate efficacy of new treatments. Thus, this specific outcome has become widely accepted as an appropriate outcome measure in COVID-19 trials. By adopting this outcome to our study, we allowed for better comparison with similar studies on the subject. In addition, the revised outcome permitted a feasible sample size.

### Strengths and limitations

Strength of our trial include the double-blinded and placebo-controlled multicenter design, few dropouts and near complete follow up. Moreover, only high-titer convalescent plasma was used in the study. Limitations included a high rate of ineligibility that may reduce the external validity as well as insufficient sample size and statistical power owing to the premature termination of the trial.

## Conclusion

In conclusion, the infusion of convalescent plasma did not influence clinical progression, survival, or length of hospitalization in patients with COVID-19 pneumonia when administered during hospital admission.

## Supplementary Information

Supplementary Information.

## Data Availability

Data will not be made available for download due to restrictions regarding privacy under the EU GDPR. Access to data may be granted on specific requestion to Thomas.benfield@regionh.dk and only in agreement with the rules and regulations of the Danish Data Protection Agency and the National Committee on Health Research Ethics.
