# Supplementary material for: A randomized placebo-controlled trial of convalescent plasma for adults hospitalized with COVID-19 pneumonia
Source: Sci Rep. 2022 Sep 30;12:16385. doi: 10.1038/s41598-022-19629-z (PMC9523654; doi:10.1038/s41598-022-19629-z)

**SUPPLEMENTAL MATERIAL**

**Supplemental table S1.**  Assumed severity distribution at day 14

|  | 14-day probability (%) | Intervention (OR = 1.6) |
| --- | --- | --- |
| Death | 10 | 6·5 |
| Hospitalized, in intensive care requiring Extracorporeal Membrane Oxygenation (ECMO) or mechanical ventilation | 10 | 7·0 |
| Hospitalized, on non-invasive ventilation or high-flow oxygen device | 4 | 3·0 |
| Hospitalized, requiring supplemental oxygen | 8 | 6·3 |
| Hospitalized, not requiring supplemental oxygen | 7 | 5·8 |
| Not hospitalized, limitation on activities and/or requiring home oxygen | 32 | 31·9 |
| Not hospitalized, no limitations on activities | 29 | 39·5 |

**Supplemental table S2.** Reported adverse events during follow-up

| **Adverse event**  **(severe adverse events not included)** | **Event description** | **Event grade** |
| --- | --- | --- |
| **Intervention group** | |  |
| No 1 | Chills | 1 |
| No 2 | Skin rash | 2 |
| No 3 | Deep venous thrombosis | 2 |
| No 4 | Decline in oxygen saturation | 1 |
| No 5 | Cough | 1 |
| No 6 | Dyspnea | 1 |
| No 7 | Pneumonia | 1 |
| No 8 | Weakness | 1 |
| No 9 | Back pain | 1 |
| No 10 | Deep venous thrombosis | 2 |
| No 12 | Bacterial infection | 2 |
| No 13 | Need of increased oxygen supply | 2 |
| No 14 | Elevated D-dimer | 1 |
| No 15 | Elevated potassium | 1 |
| No 16 | *S. aureus* bacteremia | 2 |
| No 17 | Hypoglycemia | 2 |
| No 18 | Tachycardia | 1 |
| **Placebo group** | |  |
| No 1 | Hypotension | 2 |
| No 2 | Elevated liver enzymes | 1 |
| No 3 | Diarrhea | 1 |
| No 4 | Dyspnea | 1 |
| No 5 | Loss of taste | 1 |
| No 6 | Headache | 1 |
| No 7 | Concentration problems | 1 |
| No 8 | Chills | 1 |
| No 9 | Chest pain | 1 |
| No 10 | Swollen tongue | 1 |
| No 11 | Hair loss | 1 |
| No 12 | Thrombocytopenia | 1 |

**Supplemental table S3.** Reported severe adverse events during follow-up

| **Severe Adverse Event** | **Event description** | **Event related** |
| --- | --- | --- |
| **Intervention group** | |  |
| No 1 | Respiratory distress transfer to ECCMO treatment | No |
| No 2 | Death outside of the hospital | No |
| No 3 | Death during hospitalization for covid-19 | No |
| No 4 | Death during hospitalization for covid-19 | No |
| No 5 | Hospitalization due to abdominal pain (ovarian cyst) | No |
| No 6 | Death during hospitalization for covid-19 | No |
| No 7 | Readmission due to progression of covid-19 symptoms and pneumonia | No |
| No 8 | Death during hospitalization for covid-19 | No |
| No 9 | Death during hospitalization for covid-19 | No |
| No 10 | Hospitalization due to foot ulcer | No |
| No 11 | Death during hospitalization for covid-19 | No |
| No 12 | Death during hospitalization for covid-19 | No |
| No 13 | Readmission due to progression of covid-19 symptoms | No |
| No 14 | Hospitalization due to bacterial infection | No |
| No 15 | Hospitalization due to urinary tract infection | No |
| No 16 | Death during hospitalization for covid-19 | No |
| No 17 | Death during hospitalization for covid-19 | No |
| No 18 | Death during hospitalization for covid-19 | No |
| No 19 | Death during hospitalization for covid-19 | No |
| No 20 | Death during hospitalization for covid-19 | No |
| No 21 | Death during hospitalization for covid-19 | No |
| No 22 | Death during hospitalization for covid-19 | No |
| No 23 | Readmission due to progression of covid-19 symptoms | No |
| No 24 | Transfer to intensive care unit | No |
| No 25 | Transfusion related reaction | Yes |
| **Placebo group** |  |  |
| No 1 | Death during hospitalization for covid-19 | No |
| No 2 | Hospitalization due to urinary tract infections | No |
| No 3 | Death during hospitalization for covid-19 | No |
| No 4 | Death during hospitalization for covid-19 | No |
| No 5 | Hospitalization due to delirium/ depression | No |
| No 6 | Hospitalization due to urinary tract infection | No |
| No 7 | Hospitalization due to infection and dehydration | No |
| No 8 | Death during hospitalization for covid-19 | No |

FIGURE LEGENDS

**Figure S1.** Illustration of the blinding procedure related to treatment assignment using opaque covers to conceal intravenous lines and infusion bags

**Figure S1.**


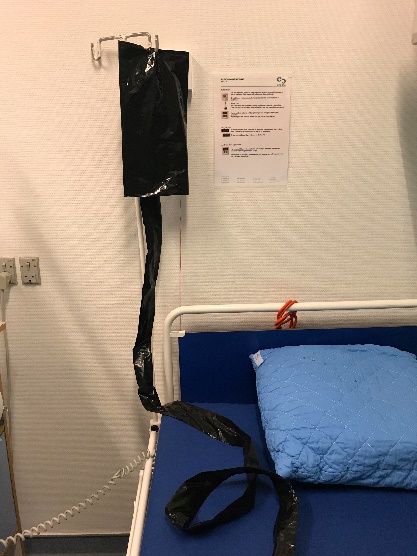

Supplement: Supplementary file 1 — Supplementary Information. [file 41598_2022_19629_MOESM1_ESM.docx]
